# Psychometric Analysis of the Rapid Eating Assessment for Participants-Short Form to Evaluate Dietary Quality in a Pre-Surgical Bariatric Population

**DOI:** 10.3390/nu15153372

**Published:** 2023-07-29

**Authors:** Daisuke Hayashi, Travis D. Masterson, Ann M. Rogers, Andrea Rigby, Melissa Butt

**Affiliations:** 1 Department of Nutritional Sciences, The Pennsylvania State University, University Park, PA 16802, USA; dbh5557@psu.edu (D.H.); travis.d.masterson@psu.edu (T.D.M.); 2 Division of Minimally Invasive and Bariatric Surgery, Department of Surgery, Penn State Milton S. Hershey Medical Center, Hershey, PA 17033, USA; arogers@pennstatehealth.psu.edu (A.M.R.); arigby@pennstatehealth.psu.edu (A.R.); 3 Department of Public Health Sciences, Penn State College of Medicine, Hershey, PA 17033, USA; 4 Department of Family and Community Medicine, Penn State College of Medicine, Hershey, PA 17033, USA

**Keywords:** dietary quality, surgical weight loss, bariatric surgery, eating behaviors, confirmatory factor analysis

## Abstract

Dietary quality and eating behaviors are essential to evaluating bariatric surgery candidates. The Rapid Eating Assessment for Participants–Short Form (REAP-S) is a previously validated measure of dietary quality suited for use in primary care. This study aimed to evaluate the psychometric properties of the REAP-S in a pre-surgical bariatric population. This study included data from one academic medical center from August 2020 to August 2022. Variables included socio-demographics, the REAP-S, mental health, and assessments of appetitive traits. Statistical methods included Cronbach’s alpha, confirmatory factor analysis (CFA), and multivariable analyses. A total of 587 adult patients were included in this analysis. The mean score for the REAP-S was 28.32 (SD: 4.02), indicative of relatively moderate dietary quality. The internal consistency of the REAP-S was moderate, with a Cronbach’s alpha of 0.65. The three-factor CFA model resulted in a comparative fit index of 0.91. Race (*p* = 0.01), body mass index (*p* = 0.01), food fussiness (*p* < 0.0001), food responsiveness (*p* = 0.005), and socially desirable responses (*p* = 0.003) were significantly associated with the total REAP-S score. Although the REAP-S’s original purpose was to assess dietary quality within a primary care population, it shows promise for application within a bariatric surgery-seeking population.

## 1. Introduction

Modern bariatric procedures (e.g., sleeve gastrectomy and Roux-en-Y gastric bypass) are currently the most effective treatments for severe obesity. These treatments promote long-term weight loss and greater glycemic control, blood pressure, and lipid profile improvements than nonsurgical treatments [1]. Successful treatment through bariatric surgery, however, requires careful medical, nutritional, and behavioral assessment and monitoring of patients by a multidisciplinary team of healthcare professionals, including determining eligibility, preparing them for surgery, as well as providing immediate and long-term post-operative care to avoid complications and weight regain [2,3]. Specifically, current guidelines by the American Society for Metabolic and Bariatric Surgery (ASMBS) highlight the importance of examining dietary patterns and potential maladaptive eating behaviors in the nutritional assessment of patients seeking bariatric surgery [4]. Recommendations to assess eating behaviors and patterns include using tools such as 24-h recalls, food diaries, structured interviews, and self-reported validated questionnaires as necessary [3].

One such nutrition assessment tool available for healthcare professionals is the Rapid Eating Assessment for Participants (REAP) [5] and its updated Short Version (REAP-S) [6]. The REAP-S was initially developed as a triage tool by primary care providers to rapidly assess the quality of a patient’s diet, focusing on the consumption of selected food groups of particular interest in managing chronic metabolic diseases [5]. The shortened instrument was first validated with a population of medical students against the Block 1998 Food Frequency Questionnaire [6] and remained a quick assessment for use in primary care to assess nutritional intake [7]. In a more recent study, the REAP-S score was significantly correlated with another well-established measure of dietary quality, the Healthy Eating Index (HEI)-2010, in a sample of 81 healthy adults [8]. That same study also found the REAP-S score to correlate significantly with the nutrient density of the participants’ diets and their plasma vitamin C concentrations. Despite showing promise as a time-efficient and low-cost measure of dietary quality for use in clinical settings, no study has since demonstrated whether the REAP-S would be an appropriate measure for use as part of the pre-surgical assessment of patients seeking bariatric surgery.

Therefore, this study aimed to evaluate the psychometric properties of the REAP-S in pre-surgical bariatric patients and explore its association with sociodemographic factors and measures of appetitive traits and mental health in this population. The primary hypothesis of this analysis is that the REAP-S would show adequate psychometric properties in a pre-surgical bariatric patient population, similar to other previously studied clinical populations. Additionally, we hypothesized that dietary quality would be significantly associated with self-reported appetitive traits and adverse mental health outcomes.

## 2. Materials and Methods

This study was conducted on a prospectively collected data repository of presurgical psychological evaluation testing administered to patients seeking treatment in a surgical weight loss program. Participants were recruited from a single academic medical center from August 2020 until August 2022. The self-reported testing was administered via REDCap [9], an online web-based application used for data collection and management throughout the data collection period. Pediatric (age under 18) patients were excluded from the analysis.

### 2.1. Measures

The REAP-S is a 16-item questionnaire that assesses the dietary quality of the respondent and has been previously validated for use in various populations [6]. The first 13 items of the assessment inquire about various eating practices, including skipping breakfast, consuming food from restaurants, consuming less than recommended servings of whole grains, fruit, vegetables, dairy, and more than the recommended servings of meat, as well as consuming foods/drinks that are high in fats and sugars. These items are rated on a three-point frequency scale of “usually/often” (score = 1), “sometimes” (score = 2), and “rarely/never” (score = 3), with some items allowing for a “not applicable” (score = 3) response. Additionally, the survey asks two questions about shopping and cooking and one item inquiring about willingness to change eating habits. For a total instrument score, the first 13 items are summed for a total score ranging from 13 (low dietary quality) to 39 (higher dietary quality). The remaining three questions were analyzed separately.

Sociodemographic variables, including age, sex, race (categorized as White and Non-White), and the highest level of educational attainment, were collected from the patient. In addition to these items, participants were asked to complete the Weight Bias Internalization Scale (WBIS) [10], Marlowe-Crowne Social Desirability Scale 13-Item Short Form C (MC-C) [11], the Adult Eating Behavior Questionnaire (AEBQ) [12], Beck Depression Inventory II (BDI-II) [13], and the Burns Anxiety Inventory (BAI) [14]. The first item of the WBIS was dropped from analyses in accordance with previous studies [15]. Additionally, the AEBQ was broken into eight appetitive traits associated with eating behaviors that were included in the models as independent variables, including enjoyment of food, food fussiness, emotional overeating, hunger, satiety responsiveness, food responsiveness, slow eating, and emotional undereating. Patients were also asked to self-report their height and weight, which were then used to calculate body mass index (BMI; kilogram/square meter).

### 2.2. Statistical Analyses

These data were analyzed in June of 2023 using SAS Version 9.4 (SAS Institute, Cary, NC, USA) with the level of significance set to 0.05. Descriptive statistics were used to characterize the sample. Internal consistency was evaluated using item-total correlations and Cronbach’s alpha. Spearman Correlation Coefficients were calculated with Fisher z transformation to obtain 95% Confidence Limits between the individual items of the REAP-S and between the REAP-S and other interested variables.

Two confirmatory factor analyses (CFA) were conducted using a single-factor and three-factor structure and a correlation structure analysis with a factor variance set to 1. The three-factor structure was determined by the study team based on a thematic review of the first 13 individual items as well as the original scale. These three factors were meal practices, MyPlate quality, and nonadherence to MyPlate. The first factor (meal practices) comprised the first two items and was grouped as a domain in the original assessment. The next factor (MyPlate quality) comprised the following five items based on the current major USDA MyPlate food groups of whole grains, fruits, vegetables, dairy, and proteins [16]. The last factor (nonadherence to MyPlate) included the final six items that was based on dietary practices contributing to a high added sugars, salt, and fat intake, which should be consumed in moderation. For both the single- and three-factor models, the root mean square error of approximation (RMSEA), Standardized Root Mean Square Residual (SRMR), and comparative fit index (CFI) were calculated and compared.

A multivariate linear regression model was built using the REAP-S and the three factors as the dependent variables. Continuous independent variables were standardized using the total sample standard deviation of the instrument. Independent variables included socially desirable responding (from MC-C), depressive symptoms (from BDI-II), anxious symptoms (from BAI), appetitive traits (from AEBQ), age, sex, race, and BMI. As this analysis included testing multiple dependent variables, Bonferroni adjustments were made to the significance level to account for multiple comparisons (α = 0.05/4 = 0.0125).

Due to the self-reported nature of this survey, missing data did occur but were minor. The percentage of missing data ranged from 0.17% to 2.05% (Table 1). Responses with missing data were excluded from analyses depending on the variables included.

## 3. Results

A total of 587 adult patients were included in this analysis (Table 1). The mean age of the sample was 42.97 years (standard deviation [SD]: 11.74), mostly female (*n* = 457; 77.99%), identified as White (*n* = 432; 74.23%), with educational attainment of high school or less (*n* = 287; 49.91%). The mean BMI was 48.08 (SD: 8.93). The mean score for the REAP-S was 28.32 (SD: 4.02; observed range: 16–38), indicative of relatively moderate dietary quality.

**Table 1:** Sample Characteristics (*n* = 587).

Variable	Total Sample	Observed Range	Total Possible Range	Missing Data N (%)
Body Mass Index, Mean (SD)	48.08 (8.93)	(35.12, 98.15)		10 (1.70%)
Age, Mean (SD)	42.97 (11.74)	(18, 72)		2 (0.34%)
Sex, N (%)				1 (0.17%)
Male	129 (22.01)			
Female	457 (77.99)			
Race, N (%)				5 (0.85%)
White	432 (74.23)			
Non-White	150 (25.77)			
Education, N (%)				12 (2.05%)
HS or less	287 (49.91)			
Some College	114 (19.83)			
Bachelors	113 (19.65)			
Graduate	61 (10.61)			
Instrument Scores				
REAP-S, Mean (SD)	28.32 (4.02)	(16, 38)	(13, 39)	2 (0.34%)
MC-C, Mean (SD)	9.37 (2.56)	(0, 13)	(0, 13)	12 (2.05%)
WBIS, Mean (SD)	4.40 (1.40)	(1, 7)	(1, 7)	4 (0.68%)
BDI-II, Mean (SD)	12.23 (9.91)	(0, 53)	(0, 63)	5 (0.85%)
BAI, Mean (SD)	14.46 (14.31)	(0, 77)	(0, 99)	6 (1.02%)
AEBQ, Mean (SD)				
Enjoyment of Food	11.58 (2.17)	(4, 15)	(3, 15)	11 (1.87%)
Food Fussiness	12.29 (4.41)	(5, 25)	(4, 20)	11 (1.87%)
Emotional Overeating	13.69 (5.06)	(5, 25)	(5, 25)	11 (1.87%)
Hunger	13.51 (3.64)	(5, 24)	(5, 25)	11 (1.87%)
Satiety Responsiveness	10.81 (2.94)	(4, 20)	(4, 20)	11 (1.87%)
Food Responsiveness	11.26 (3.21)	(4, 20)	(5, 25)	11 (1.87%)
Slow Eating	10.64 (3.71)	(4, 20)	(4, 20)	11 (1.87%)
Emotional Undereating	13.35 (4.53)	(5, 25)	(5, 25)	11 (1.87%)

SD: Standard Deviation; HS: High School; REAP-S: Rapid Eating Assessment for Participants—Short Form; MC-C: Marlowe-Crowne Social Desirability Scale 13-Item Short Form C; WBIS: Weight Bias Internalization Scale; BDI-II: Beck Depressive Inventory—II; BAI: Burns Anxiety Inventory; AEBQ: Adult Eating Behavior Questionnaire.

By frequency (Table 2), consumption of more than 8 ounces of animal protein (item 7) and adding butter, margarine, or oil to foods (item 11) had the highest percentage of “usually” responses (*n* = 191; 32.65%). Additionally, 27.18% (*n* = 129) of respondents reported “usually” skipping breakfast (item 1). Among the other MyPlate categories, consumption of less than two servings of dairy (item 6) and fruits (item 4) resulted in the highest frequencies of occurrence, with 152 (25.98%) and 150 (25.64%), respectively. Overall, the internal consistency of the REAP-S was moderate, with a Cronbach’s α of 0.65. Of all the items, item 3 (servings of whole grains) had the lowest item-total correlation (0.02), followed by item 6 (servings of dairy; 0.18).

**Figure 1:**
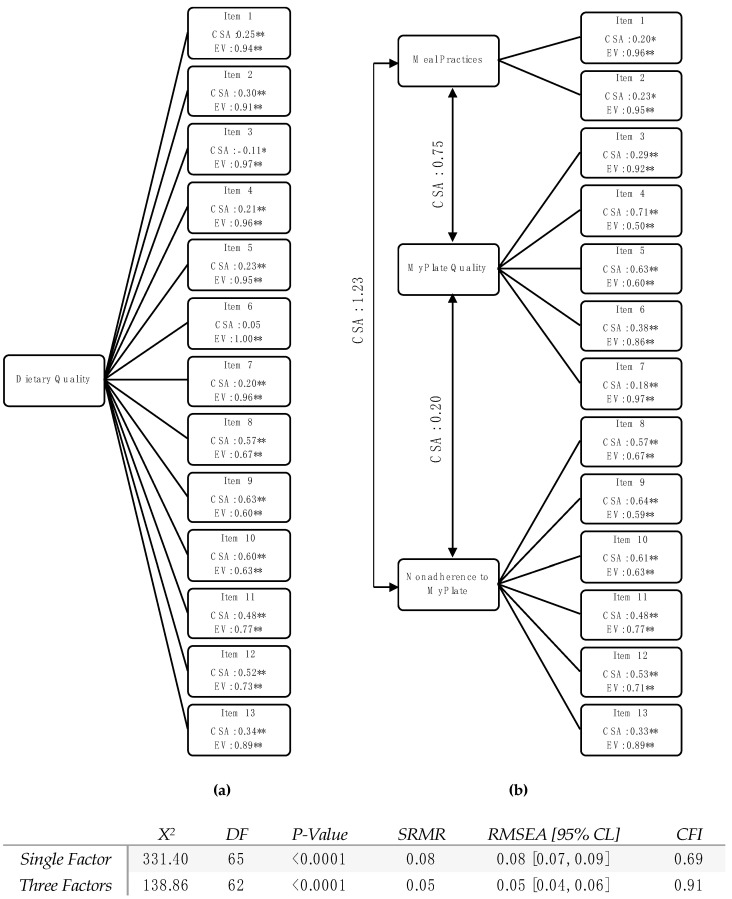
Confirmatory Factor Analysis of the REAP-S. Path Diagram for single (**a**) and three (**b**) factor structures. CSA: Correlation Structure Analysis; EV: Error Variance DF: Degrees of Freedom; SRMR: Standardized Root Mean Square Residual; RMSEA: Root Mean Square Error of Approximation; and CFI: Comparative Fit Index; ** significant < 0.01; * significant < 0.05.

Two CFA models were built to compare the latent factor structure of the first 13 items of the REAP-S (Figure 1a,b). In the single-factor model, two items, in particular, had lower factor loadings, with one being non-significant (item 6). All factor loadings were statistically significant in the three-factor model. Between the two CFA models, the three-factor model demonstrated a better fit to the data than the single-factor model (Figure 1). The SRMR of the three-factor model was 0.05 compared to an SRMR of 0.08 for the single-factor model. Additionally, the RMSEA was notably improved in the three-factor model with a value of 0.05 (95%CL [0.04, 0.06]) compared to 0.08 for the single-factor model. The CFI of the three-factor model also indicated a good fit with a value of 0.91, compared to 0.69 for the single-factor model.

In terms of univariable associations (Table 3), the REAP-S score was positively and weakly to fairly associated with socially desirable responding (0.23), satiety responsiveness (0.14), and slow eating (0.12), indicating that higher dietary quality scores were associated with higher scores for the variables mentioned above. The REAP-S score was also positively and weakly associated with non-White race (0.09). Additionally, hunger (−0.10), BMI (−0.14), internalized weight bias (−0.14), enjoyment of food (−0.18), food fussiness (−0.19), emotional overeating (−0.19), food responsiveness (−0.20), depressive symptoms (−0.20), and anxious symptoms (−0.22) were inversely and weakly to fairly correlated with the REAP-S; indicating that lower dietary scores were associated with higher scores for these variables.

When all covariates were included in linear regression models (Table 3), several factors were identified as statistically significant. When considering the total REAP-S score, food fussiness [β: −0.67 (0.16); <0.0001] and food responsiveness [β: −0.68 (0.24); 0.005] were significantly associated with the total score. Further, while anxious symptoms overall were not significantly associated with the total score, participants with severe symptoms of anxiety (compared to no symptoms) had significantly lower REAP-S scores [β: −1.97 (0.78); 0.01]. The inverse associations between REAP-S scores and food fussiness, food responsiveness, and severe anxiety scores demonstrate that increases in pathology were associated with lower dietary quality.

When exploring these associations across the three latent factors, there were notable differences. Age [0.11 (0.04); 0.01] and hunger [0.18 (0.06); 0.001] were significantly associated with scores on the meal practices factor; there were no significant associations with the MyPlate Quality factor. Food fussiness [−0.49 (0.11); <0.0001] and socially desirable responding [0.37 (0.12); 0.003] were associated with scores on the nonadherence to MyPlate factor.

## 4. Discussion

This study was the first to evaluate the psychometric properties of the REAP-S among individuals with obesity seeking bariatric surgery. It was also the first study to examine the relationship between REAP-S scores as a measure of dietary quality and sociodemographic factors, assessments of mental health, and assessments of appetitive traits in this population. Our results show that the REAP-S had moderate internal consistency, and its score was negatively associated with food fussiness, food responsiveness, male sex, and non-white race/ethnicity. Additionally, we showed that a three-factor structure of the REAP-S would be more appropriate than a single-factor one.

Although previous studies have assessed the validity of the REAP-S against other measures of dietary quality and found moderate to high correlations [6,8], no studies, to our knowledge, have assessed its internal consistency. The moderate internal consistency (Cronbach’s α = 0.65) in our study could suggest that, although the different items in the REAP-S are related to the construct of healthy eating to some extent, the individual dietary behaviors and practices assessed by this tool are not perfectly consistent among themselves [17]. Considering our population, this notion can be rather intuitive since a candidate for bariatric surgery who struggles with severe obesity might often add butter or margarine to their dishes and consume deep-fried foods (behaviors associated with low dietary quality) but still consistently meet their daily vegetable and fruit intake recommendations (factors associated with high dietary quality). In this case, a Cronbach’s α too close to 1 with a near-perfect correlation between different items would be undesirable for a tool that assesses dietary quality, as it could mean that the different questions in a tool are redundant and fail to capture the wide range of possible behaviors that individuals might experience with their dietary practices [17]. Likewise, the opposite scenario, in which a tool that assesses dietary quality has too low internal consistency, could mean that its items are completely unrelated.

This study also was the first one to our knowledge to conduct a CFA to determine the CFI of a single- and a three-factor structure of the REAP-S. These analyses pointed to a better-fit index for the three-factor structure, with a CFI of 0.91 versus 0.69 for the single-factor structure. This finding suggests that, for our population of interest, dividing the first 13 items of the REAP-S into three subdomains (which we labeled meal practices, MyPlate quality, and nonadherence to MyPlate) yields a better fit than a single factor. The three-factor structure is also supported by the more moderate Cronbach’s α that was calculated based on all 13 items. The lower value for internal consistency and lower CFI for the single-factor structure suggest that this assessment does not measure a single unified dietary concept [17,18].

The significant associations we identified between the total REAP-S score and sociodemographic characteristics, namely race and sex, are consistent with previous population-based data indicating that the dietary quality of Americans differs according to sex and race [19]. In that study, which analyzed dietary quality using the HEI 2005 based on NHANES data from 2003 to 2004, women showed a higher dietary quality than men. In a more recent study that examined food frequency questionnaire data from 155,331 adults across 35 US states, non-White participants displayed significant differences in dietary quality when compared to White responders, with a higher risk of poor diet quality among Black participants, and a lower risk between Hispanic/Latino, Asian, Native Hawaiian, and Pacific Islander participants [20]. Although these were population-based studies and did not focus on a population of bariatric surgery candidates, these findings could suggest that assessing dietary quality using the REAP-S could capture differences in dietary quality across demographic groups in settings where time is scarce and a shorter dietary assessment tool could be particularly useful. Similarly, our study’s negative association between BMI and total REAP-S is similar to previous findings of a systematic review that diet quality assessed using the HEI is inversely associated with body weight [21]. Moreover, in a previous study, an improvement in REAP-S score was accompanied by reduced intake of energy fat, saturated fat, sodium, sugar, and alcohol after a 12-week multi-component weight loss intervention for older adults with obesity, along with decreased BMI and increased fiber intake [22]. These findings reinforce the potential value of REAP-S as a triage and monitoring tool in weight management.

The AEBQ has previously been shown as a valid measure of appetitive traits in bariatric candidates, which can help professionals to identify and discuss determinants of potentially maladaptive eating behaviors with patients [23]. In our study, food fussiness (i.e., avoidance of unfamiliar foods and a narrower selection of foods enjoyed) and food responsiveness (i.e., eagerness to eat upon exposure to food cues) were inversely correlated with dietary quality assessed by the REAP-S. This finding is particularly interesting considering that food fussiness and responsiveness are subscales associated with avoidance and approach towards food, respectively. This association between lower dietary quality and appetitive traits indicating increased approach and avoidance tendencies toward foods is consistent with the ambivalence model of craving, in which the competing desires to consume and not consume foods are associated with a range of maladaptive eating behaviors, particularly binge-eating, which often applies to individuals with obesity and can contribute to a lower dietary quality [24,25]. Another possible explanation is that the concomitant presence of increased reactivity to food cues and lack of openness to try new foods could contribute to a higher intake of highly palatable foods, including sweets, soda, and salty snacks, and a lower intake of fruits and vegetables, all of which contribute to a lower REAP-S score.

Socially desirable responding, on the other hand, was positively correlated with a higher total REAP-S score and, more specifically, with the Dietary Practices subdomain identified in our CFA. A previous study reported that patients undergoing preoperative assessment for bariatric surgery underreported symptoms of psychopathology compared to clinical evaluations by a licensed psychologist and that self-reported measures of psychopathology, disordered eating, and quality of life were all associated with social desirability scores [26]. Our current findings could indicate that bariatric surgery candidates could also be prone to the effects of this form of response bias or impression management when reporting their eating behaviors, which could lead to underreporting unhealthy eating behaviors that would lead to a lower score of diet quality. This interpretation is particularly likely as patients could be concerned that being deemed a good candidate for surgery is contingent upon previous attempts to improve their diet without surgery. This phenomenon is consistent with previous studies that have noted how forms of impression management, particularly, impact the reporting of adverse psychopathology, another barrier that may be perceived as undesirable in a pre-surgical bariatric population [27,28].

Our psychometric data show that the REAP-S is a promising rapid triage tool for pre-surgical bariatric patients regarding dietary quality. However, there are important bariatric-specific considerations related to this tool that should be accounted for by researchers and healthcare professionals when employing it, and that could indicate a need for future adaptation of the REAP-S for this population. Particularly, some of the 13 items that compose the score of dietary quality and the way they are scored in this tool might not be compatible with evidence-based recommendations for the medical nutrition therapy of bariatric patients. For instance, although eating more than 8 ounces a day of animal sources of protein (including lean ones) contributes to a lower REAP-S score, it is often recommended that pre- and post-bariatric patients consume foods or supplements rich in proteins over those rich in carbohydrates or fats to avoid protein deficiency and promote satiety [29,30,31]. Additional considerations should be made to other items that compose the REAP-S score, which can be of difficult adherence by patients both immediately following surgery as well as for long-term follow-up due to their reduced stomach capacity and common complaints about gastrointestinal issues [32]; however, that was not the immediate focus of this particular study.

### 4.1. Strengths and Limitations

This study has some noteworthy limitations. For instance, our results may have limited generalizability across bariatric surgery candidates, as data were collected in a single university hospital. Another limitation is the lack of validity measures for REAP-S within this population, which could be investigated in future studies as convergent validity with other common measures of dietary quality, including the HEI. Additionally, as evident by the associations of the REAP-S with socially desirable responding, response bias or impression management may impact these findings. Lastly, the cross-sectional design of our study that includes only participants before bariatric surgery limits its applicability to postoperative patients. Future studies should examine changes in REAP-S scores before and after bariatric surgery to further investigate its promise and pitfalls when used for rapid dietary assessment in these populations.

Despite these limitations, this is the first study to conduct a psychometric analysis of the REAP-S within a sample of bariatric surgery candidates and to explore the associations between dietary scores assessed with REAP-S and appetitive traits, sociodemographic factors, self-reported mental health, and demographic factors. Furthermore, nearly one-half of the participants included in this study had educational attainment of high school or below, which made it particularly well-suited to test the performance of the REAP-S among bariatric patients since this tool was initially developed for use in primary care with participants of limited educational background.

### 4.2. Clinical Implications

These data demonstrate the psychometric properties and justification for using the REAP-S in a clinical pre-surgical bariatric population and that this tool may offer a time and cost-efficient alternative for assessing dietary quality. Given the nuances of the recommended dietary practices for this population, some caution should be exercised when interpreting the overall score, particularly regarding protein consumption. Despite this, the REAP-S is a short, 13-item instrument that can be quickly administered in a clinical setting to understand better the baseline dietary practices of patients regarding three particular factors: meal practices, MyPlate quality, and nonadherence to MyPlate as well as observe a change in dietary quality and practices when administered over time.

## 5. Conclusions

Based on its psychometric properties, the REAP-S shows promise for application within a bariatric surgery-seeking population as a time-efficient assessment of dietary quality and behaviors. However, some elements of the instrument may require additional considerations for a pre-surgical bariatric population to align with standard bariatric dietary recommendations. Future research should assess the convergent validity of the REAP-S among pre-surgical bariatric patients against other measures of dietary quality, such as the HEI, and consider adapting this tool for use in post-operative patient monitoring of dietary quality.

## Figures and Tables

**Table 2:** Internal Consistency of the REAP-S (*n* = 585).

	Response Frequency N (%)			Single-Factor	
	Usually	Sometimes	Never/NA	Item-Total Correlation	Alpha If Removed
Item 1. Skip Breakfast	159 (27.18)	219 (37.44)	207 (35.38)	0.23	0.64
Item 2. Eat 4+ meals out	44 (7.52)	158 (27.01)	383 (65.47)	0.24	0.64
Item 3. <2 servings of whole grains	130 (22.22)	306 (52.31)	149 (25.47)	0.02	0.67
Item 4. <2 servings of fruit	150 (25.64)	295 (50.43)	140 (23.93)	0.34	0.62
Item 5. <2 servings of vegetables	112 (19.15)	292 (46.91)	181 (30.94)	0.33	0.62
Item 6. <2 servings of dairy	152 (25.98)	249 (42.56)	184 (31.45)	0.18	0.65
Item 7. >8 oz of animal protein	191 (32.65)	254 (43.42)	140 (23.93)	0.21	0.64
Item 8. Use regular processed meats	79 (13.50)	251 (42.91)	255 (43.59)	0.39	0.61
Item 9. Eat fried foods	77 (13.16)	297 (50.77)	211 (36.07)	0.41	0.61
Item 10. Eat regular snack foods > low-fat	105 (17.95)	279 (47.69)	201 (34.36)	0.39	0.61
Item 11. Add butter, margarine, or oil	191 (32.65)	257 (43.93)	137 (23.42)	0.33	0.62
Item 12. Eat sweets	69 (11.79)	233 (39.83)	283 (48.38)	0.33	0.62
Item 13. >16 oz of non-diet soda or juice	64 (10.94)	111 (18.97)	410 (70.09)	0.28	0.63
Cronbach’s Alpha					0.65

**Table 3:** Associations between eating behaviors, mental health, and the REAP-S.

	Univariable Associations	Multivariate Associations			
Independent Variables	Spearman Correlation Coefficient (95%CL)	Total REAP-S	Factor 1: Meal Practices	Factor 2: MyPlate Quality	Factor 3: Nonadherence to MyPlate
Age	0.08 [−0.01, 0.16]	0.30 (0.17); 0.07	0.11 (0.04); 0.01 **	0.16 (0.09); 0.08	−0.03 (0.11); 0.81
Sex (Ref: Female)	0.02 [−0.06, 0.10]	−0.19 (0.39); 0.64	−0.05 (0.11); 0.64	−0.18 (0.22); 0.42	0.04 (0.26); 0.87
Race (Ref: White)	0.09 [0.01, 0.17] *	−0.78 (0.40); 0.05	−0.25 (0.11); 0.02 *	−0.12 (0.22); 0.59	−0.41 (0.26); 0.12
BMI	−0.14 [−0.22, −0.06] *	−0.40 (0.16); 0.01 *	−0.08 (0.04); 0.07	−0.17 (0.09); 0.06	−0.15 (0.11); 0.17
MC-C	0.23 [0.15, 0.31] *	0.42 (0.18); 0.02 *	0.03 (0.05); 0.54	0.02 (0.10); 0.82	0.37 (0.12); 0.003 **
WBIS	−0.14 [−0.22, −0.06] *	0.10 (0.21); 0.62	0.03 (0.06); 0.60	−0.02 (0.12); 0.90	0.09 (0.14); 0.51
AEBQ					
Enjoyment of Food	−0.18 [−0.26, −0.10] *	−0.17 (0.19); 0.38	−0.05 (0.05); 0.34	0.06 (0.11); 0.56	−0.19 (0.13); 0.15
Food Fussiness	−0.19 [−0.27, −0.11] *	−0.67 (0.16); <0.0001 **	−0.03 (0.04); 0.54	−0.15 (0.09); 0.10	−0.49 (0.11); <0.0001 **
Emotional Overeating	−0.19 [−0.27, −0.11] *	−0.32 (0.21); 0.14	−0.06 (0.06); 0.29	0.06 (0.12); 0.64	−0.31 (0.14); 0.03 *
Hunger	−0.10 [−0.18, −0.02] *	0.44 (0.21); 0.03 *	0.18 (0.06); 0.001 **	0.18 (0.12); 0.12	0.08 (0.14); 0.54
Satiety Responsiveness	0.14 [0.06, 0.22] *	0.08 (0.20); 0.67	−0.08 (0.05); 0.13	0.06 (0.11); 0.61	0.11 (0.13); 0.41
Food Responsiveness	−0.20 [−0.28, −0.12] *	−0.68 (0.24); 0.005 **	−0.10 (0.06); 0.13	−0.32 (0.14); 0.02 *	−0.26 (0.16); 0.10
Slow Eating	0.12 [0.04, 0.20] *	0.20 (0.18); 0.27	0.08 (0.05); 0.08	−0.01 (0.10); 0.93	0.12 (0.12); 0.30
Emotional Undereating	0.02 [−0.07, 0.10]	−0.09 (0.17); 0.60	−0.03 (0.05); 0.55	0.03 (0.10); 0.76	−0.09 (0.11); 0.41
BDI-II	−0.20 [−0.27, −0.12] *	*p* = 0.86	*p* = 0.19	*p* = 0.35	*p* = 0.50
Mild vs. None		−0.29 (0.50); 0.56	−0.17 (0.13); 0.20	0.18 (0.28); 0.52	−0.30 (0.33); 0.36
Moderate vs. None		0.19 (0.62); 0.75	−0.23 (0.17); 0.17	0.63 (0.35); 0.07	−0.21 (0.41); 0.61
Severe vs. None		0.29 (0.89); 0.75	−0.48 (0.24); 0.04 *	0.35 (0.50); 0.49	0.43 (0.59); 0.47
BAI	−0.22 [−0.29, −0.14] *	*p* = 0.15	*p* = 0.63	*p* = 0.18	*p* = 0.24
Minimal vs. None		−0.40 (0.46); 0.38	−0.10 (0.12); 0.42	−0.32 (0.26); 0.22	0.01 (0.30); 0.97
Mild vs. None		−0.62 (0.50); 0.22	−0.18 (0.13); 0.17	−0.56 (0.28); 0.05 *	0.12 (0.33); 0.72
Moderate vs. None		−0.39 (0.66); 0.56	−0.24 (0.18); 0.18	−0.67 (0.37); 0.07	0.52 (0.44); 0.23
Severe vs. None		−1.97 (0.78); 0.01 **	−0.31 (0.21); 0.14	−1.06 (0.44); 0.02 *	−0.61 (0.51); 0.24
Extreme vs. None		−2.39 (1.20); 0.05 *	−0.07 (0.32); 0.83	−1.29 (0.67); 0.06	−1.04 (0.79); 0.19

For univariable models: Spearman rho values were calculated between the REAP-S total score and listed covariates. * Significant at α < 0.05. For multivariate models: all continuous independent variables were standardized using the sample standard deviation and are presented as β(StdErr); *p*-Value. As all continuous variables were standardized using the sample SD, parameter estimates (β) can be interpreted as the expected change in REAP-S score with each increase in SD of the independent variable. Likewise, for ordinal variables (such as depressive and anxious symptoms), parameter estimates can be interpreted as the expected change in REAP-S score compared to the reference group. * Significant at α < 0.05; ** Significant at α < 0.0125. CL: Confidence Limits; BMI: Body Mass Index (kg/m^2^); MC-C: Marlowe-Crowne Social Desirability Scale 13-Item Short Form C; WBIS: Weight Bias Internalization Scale; REAP-S: Rapid Eating Assessment for Participants-Short Form; BDI-II: Beck Depressive Inventory—II; BAI: Burns Anxiety Inventory.

## Data Availability

The data presented in this study are available on request from the corresponding author. The data are not publicly available due to regulatory and privacy concerns.
